# Assessment of variation in B-cell receptor heavy chain repertoire in patients with end-stage renal disease by high-throughput sequencing

**DOI:** 10.1080/0886022X.2018.1487862

**Published:** 2019-01-15

**Authors:** Lei Wang, Yong Dai, Song Liu, Liusheng Lai, Qiang Yan, Huaizhou Chen, Jiaxing Zhang, Weiguo Sui

**Affiliations:** a The First School of Clinical Medicine, Southern Medical University, Guangzhou, Guangdong, China;; b Nephrology Department of Guilin No.181 Hospital, Guangxi Key Laboratory of Metabolic Diseases Research, Guilin Key Laboratory of Kidney Diseases Research, Guilin, Guangxi, China;; c Clinical Medical Research Center of the Second Clinical Medical College, Jinan University, Shenzhen People's Hospital, Shenzhen, Guangdong, China

**Keywords:** End-stage renal disease, B-cell response, immune repertoire, next generation sequencing, B-cell receptor

## Abstract

**Background/Aims:** End-stage renal disease (ESRD), characterized by progressive loss of rental function during the disease course, has been reported to be correlated with immune dysregulation. To date, a majority of previous studies on immune response to ESRD have been focused on the T-cell response. This prospective study was to assess the B-cell receptor (BCR) heavy chain repertoire in ESRD patients.

**Materials and methods:** A total of 10 ESRD patients and six healthy controls were prospectively enrolled in this study. BCR immunoglobulin heavy chain (IGH) repertoire in the peripheral blood from ESRD patients and healthy individuals were analyzed by means of next generation sequencing (NGS) in combination with multiplex PCR, Illumina sequencing, and the international ImMunoGeneTics database (IMGT).

**Results:** Abnormal BCR complementary-determining region 3 (CDR3) sequences were identified in relation to ESRD. We also found that the degree of the B-cell clonal expansion in the ESRD group was significantly greater than that in the control group (*p* < .05), whereas the distributions of BCR CDR3, V, D, J, and V–J gene segments were comparable between the ESRD and control groups. T-test for analysis of the distribution ratio of the V, D, J, and V–J genes revealed five up-regulated genes and nine down-regulated genes associated with ESRD, and there were significant differences between the ESRD and control groups (*p* < .05).

**Conclusions:** We have provided a successful approach to analyzing peripheral B-cell repertoire in ESRD patients, and the results suggest a direct correlation between the BCR repertoire and ESRD. The ESRD-specific BCR CDR3 sequences may hold promise for potentially therapeutic benefit.

## Introduction

Chronic kidney disease (CKD) is characterized by a progressive loss of renal function and its prevalence remains on the rise globally as a result of increasing patients with diabetes and hypertension, which have been identified as major risk factors for CKD. The first decade of the 21st century witnessed advance in an evaluation, classification, and stratification of CKD based on the National Kidney Foundation–Kidney Disease Outcomes Quality Initiative (NKF-KDOQI) guideline [1], which greatly facilitated the diagnosis and management of patients with CKD. However, a large proportion of CKD patients may progress to develop eventually end-stage renal disease (ESRD), also known as end-stage kidney disease. In fact, the incidence rates of ESRD in patients with diabetes and hypertension are increasing. To date, the pathogenic mechanisms underlying the development and course of ESRD remain elusive. Recent studies have demonstrated that elevated levels of albuminuria and reduced levels of glomerular filtration rate (GFR) were independently related to mortality, cardiovascular events, and the rate of ESRD [2]. Kidney failure was defined by presence of either one of the following two criteria: (1) GFR less than 15 mL/min per 1.73 m [2,3], which is usually accompanied by clinical signs and symptoms of uremia in most ESRD cases; or (2) a need for starting kidney replacement therapy (dialysis or transplantation). Actually, kidney failure is not synonymous with ESRD, in the United States, ‘End-stage renal disease’ represents an administrative term, indicating that patients are in need of receiving treatment with dialysis or renal transplantation, and that the condition is eligible for payment with health care.

As an additional characteristic, immune activation featured by systemic inflammation and immune impairment [4,5] was reported to be simultaneously associated with ESRD. It has been well known that human peripheral lymphocytes mainly include T and B-cells. A variety of T cells (e.g., memory T cells, Th1/Th2 cells, and regulatory T cells) were previously found in relation to altered immunity in ESRD patients as compared with control individuals [6,7]. B cells account for nearly 20% of the total peripheral lymphocytes, and they play a critical role in antibody production and in mediation of immune response. A number of previous studies [8–12] demonstrated that B lymphocytes significantly reduced in adult and children patients with ESRD. Furthermore, it has been reported [9] that CD5 + inherent B cells and CD27 + memory B cells were significantly decreased in children patients with chronic renal failure compared with controls. In a recent study, Pahl et al. [8] indicated that several subtypes of B cells were significantly reduced in ESRD adult patients as compared to control individuals. However, the exact role for B cells in immune deficit of the ESRD patients remains largely unknown.

An effective B-cell response largely depends on all B cells with distinct B-cell receptors (BCRs), which possess the capability of specifically recognizing and binding antigens. BCRs are structurally connected by paired heavy and light chains, being consist of variable region (V), consistent region (C, heavy chain only), and variable region (V). It has been well-documented that V contains VH and VL domains, which are composed of three complementary determining regions (CDR1, CDR2, and CDR3). Of these, CDR3 is the most variable region in the BCR genes and plays a critical role in the B-cell response to identify the original, and jointly determine the BCR antigen specificity [13]. Immunoglobulin heavy chains (IGH) are encoded by recombined Variable (V), Diversity (D), and Joining (J) genes (IGHV, IGHD, and IHGJ), while VJ rearrangements of kappa and lambda chain V genes (IGKV, IGLV) and J genes (IGKJ, IGLJ) encode the immunoglobulin light chains (IGL) [14,15]. The diversity of BCRs is primarily created by the recombination of V, D (heavy chain only), and J gene segments, and further generated by nucleotide mutations including deletions and additions in the gene segments. Moreover, upon activation, B cells undergo diversification step for selecting the B cells with high affinity for antigens.

In the present study, we aimed to assess the diversity of the B-cell immune in ESRD patients compared to normal controls by sequencing the BCR CDR3 using multiplex-PCR followed by high throughput sequencing (HTS). In addition, we intended to explore the association between the immune repertoire and ESRD. The findings gained through this study may identify the disease-specific CDR3 sequences, which may hold promise as biomarker of this disease, or potential in the development of novel approaches for prevention and treatment of ESRD.

## Materials and methods

### Human subjects and clinical samples

A total of 10 ESRD patients and six healthy individuals were prospectively enrolled in this study between January and September 2013 at the181st Hospital of Guilin, China.

In this study, ESRD was diagnosed in accordance to the NKF-KDOQI guideline, pathologically confirmed, and clinically defined as follows: (1) GFR less than 15 mL/min per 1.73 m^2^, which was accompanied by signs and symptoms of uremia in most cases; or (2) a need for initiating a kidney replacement therapy (e.g., dialysis or transplantation). The ESRD patients had a mean age of 34.12 ± 11.33 years, ranging from 20 to 54 years. The normal control individuals matched for age, gender, and ethnicity without exhibiting any laboratory and clinical signs for immunological disorder or chronic kidney disease. Clinical data including serum creatinine levels, GFR, and levels of albuminuria were recorded. Peripheral blood samples were collected from the ESRD patients and healthy donors, and PBMCs were separated.

This study was performed in compliance with the Declaration of Helsinki. All participants had given their written informed consent, and this prospective study protocol was reviewed and approved by the Ethics Committee of the181st Hospital of Guilin.

### Multiplex-PCR amplification of the BCR CDR3 region

As defined based on the criteria of the International Immuno-genetics collaboration, the BCR CDR3 represents the region starting with the second conserved cysteine encoded by the 51 portion of the V gene segment and ending with the conserved phenylalanine encoded by the six portion of the J gene segment. To create a template library for subsequent analysis with Genome Analyzer, a multiplex-PCR was designed and performed through which rearranged BCR CDR3 regions from the genomic DNA were amplified using the 12 forward primers with each specific to a functional BCR-V segment, and four reverse primers with each specific to a BCR-J segment. Both forward and reverse primers at their 5′ ends harbor the universal sequences for forward and reverse primers, respectively, which are compatible with GA2 cluster station solid-phase PCR. After completion of multiplex-PCR amplification and agarose gel electrophoresis selection, the PCR products were subsequently purified using QIA quick PCR Purification Kit. The final library was quantitated by determining the average molecule length using the Agilent 2100 bio-analyzer instrument (Agilent DNA 1000 Reagents, Low DNA Mass™ ladder, Life Technologies, Carlsbad, CA, USA) and by real-time quantitative PCR (QPCR) (TaqMan Probe, TaqMan FastStart Probe Master Mix, Roche, Basel, Switzerland). The libraries were amplified with c-Bot to generate the cluster on the flow cell, and the amplified flow cell was pair-end sequenced on an Illumina MiSeq instrument (Illumina, San Diego, CA, USA), with a read length of 100 as the most frequently used sequencing strategy. To ensure the quality of information analysis, the raw reads which contained low quality sequence and adaptor sequence were filtered in this study, after which the clean reads which can be used for subsequent data analysis were obtained. Data processing was described as follows: (1) removal of the reads with adaptor; (2) removal of the N reads (N represents unable to determine the base information) with proportion greater than 10%; (3) removal of the low-quality reads with quality value sQ <= 5 bases of accounts for more than 50% of the entire read.

### Data analysis

During the development of B lymphocytes, high diversity of the BCRs CDR3 were introduced by the rearrangement of the V, D, and J gene fragments, random addition or deletion of nucleotides within the V–J fragments or between the V-D-J fragments. Therefore, we can assess the diversity of B lymphocyte by the length of CDR3. The CDR3 lengths of healthy individuals were normally distributed compared to those of ESRD patients. We also systematically analyzed frequency usage of V, D, J, and V–J gene segments, using the T test of BCR H chain V, D, J, and V–J fragments, through which up-regulated genes and down-regulated genes were identified. In this study, we evaluated the diversity of the BCR repertoire with Simpson index of diversity (Ds) [16] and the Shannon–Wiener index (H’) [17].

### Statistical analysis

The analysis of statistical difference between groups was performed using the Mann–Whitney test as this study had a relatively small sample size. *p* values less than .05 were considered significant.

## Results

### Characteristics of the BCR H chain CDR3 sequences in ESRD

HTS was conducted to capture a high resolution of the nucleotide (nt) and amino acid (aa) sequences of the BCR H chain CDR3 region of the B cells from the peripheral blood in ten ESRD patients and six normal control individuals. We obtained an average number of 12,243,860.3 reads in the six healthy individuals and 14,266,181.6 reads in the 10 ESRD patients, as Sequenced Reads or Raw Reads, which contained low quality sequences and adaptor sequences, and subsequently underwent filtration in order to meet the quality requirements for further data analysis. After data integration of the samples, we obtained an average of 10,674,277.8 clean reads in the control group and 11,537,754.7 in the ESRD group. The total reads sequences, BCR sequences, in-frame sequences, total IGH CDR3 sequences, unique CDR3 nt sequences, unique CDR3 aa sequences, highly enpended clone (HEC) number, and HEC ratio were shown in Table 1, in which HEC was defined as the amount of a CDR3 sequence greater than 0.1% of the total amount of CDR3.

**Table 1.** BCR H Chain CDR3 repertoire sequence statistics.

Sample	Total reads	BCR sequences	In-frame sequences	Total IGH CDR3 sequences	Unique CDR3 nt sequences	Unique CDR3 aa sequences	Highly enpended clone number
NC-1	15410305	14967142	13317137	12584061	414914	292378	0
NC-2	15517826	14921481	13016806	12249709	391751	276673	1
NC-3	14022700	13180574	11415501	10600111	260471	181219	4
NC-4	537114	522114	465901	439959	43655	30075	2
NC-5	14696122	14273468	12483863	11778081	392836	279047	2
NC-6	13279095	12891492	11231688	10575248	432519	316507	0
RAR-1	16553849	15945885	13936412	12922736	337155	240062	1
RAR-2	15138380	13642649	11941469	10696076	149206	101208	466
RAR-3	15996968	15165256	13371043	12608164	338183	237091	0
RAR-4	15716465	15101141	13308836	12636581	471490	331745	5
RAR-5	14557579	13305361	11558377	10666652	175465	119315	317
RAR-6	14805946	13246734	11389436	10274739	178664	123120	301
RAR-7	11513495	10835160	9262966	8524525	273118	194799	10
RAR-8	13161753	12613486	11272006	10652390	422999	306882	4
RAR-9	13781818	13340650	11717325	10981099	472410	344106	1
RAR-10	11435563	10739801	9515824	8916519	380644	277004	1

### Comparative analysis of the diversity of B cells between the ESRD and control groups

**Figure 1.**
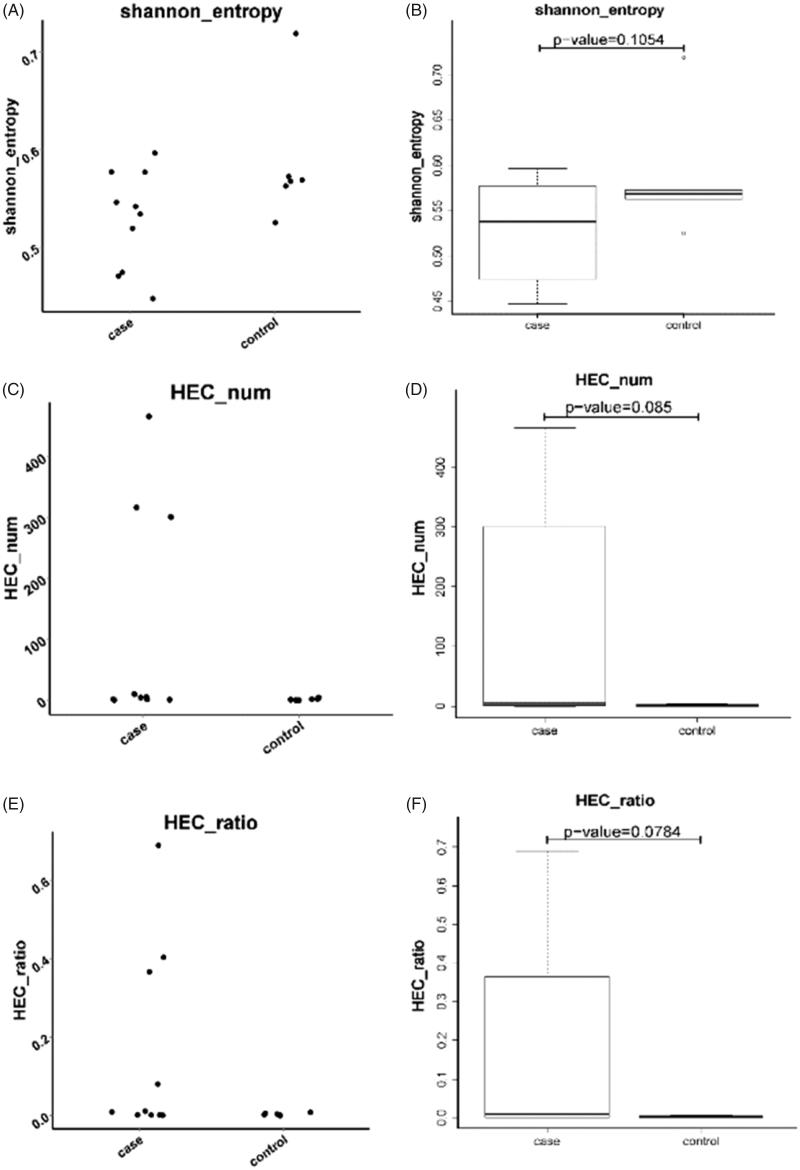
Comparison of the diversity of B cells between the ESRD and control groups. Shannon entropy distribution index were analyzed in ESRD (end-stage renal disease) patients versus healthy control individuals. (A) Shannon entropy’s scatterplot of the ESRD (end-stage renal disease) group and control group; (B) Shannon entropy’s Boxplots of the ESRD (end-stage renal disease) group versus control group. *p* = .1054 differs from control group; (C) Scatterplot of HEC in the ESRD (end-stage renal disease) patient group and healthy control group; (D) Box plot of HEC (highly enpended clone) in the ESRD (end-stage renal disease) patient group and healthy control group. *p* value was 0.085 compared with the healthy control group; (E) Scatterplot of HEC ratios of the ESRD (end-stage renal disease) group and control group; (F) Box plot of HEC (highly enpended clone) ratios of the two groups. The ESRD (end-stage renal disease) patient group exhibited significantly skewed distribution, whereas the normal control group displayed substantially normal distribution. *p* = .0784 differs from control group.

To quantitatively assess the diversity of B cells, normalized Shannon entropy index ranging from 0 to 1 was used, in which 1 represents the highest diversity and 0 indicates the lowest diversity. As shown in Figure 1, the ESRD patient group exhibited more dispersed values of Shannon entropy index with median value of 0.54 and substantially skewed distribution in contrast to the healthy control group, which presented a normal distribution with median value of 0.57. However, the difference of the Shannon entropy index between the two groups was not statistically significant (*p* > .05). In this study, we defined HEC as the expression of a CDR3 sequences greater than 0.1% of the total CDR3 sequences. HEC in the ESRD group was significantly higher than the normal control group (*p* < .05), suggesting that ESRD patients may have abnormal CDR3 sequences as amplified by PCR. It was noted that differences in the proportion of the view between the two groups were observed, but were not statistically significant (*p* > .05).

### Comparison of the CDR3 length distributions

With two-dimensional Gaussian curve fitting, we obtained the distribution of CDR3 lengths of the ESRD group (A2A, A4A, A5A, A7A, A8A, A9A, R1A, R6A, R8A, and R10A) and the control group (K1A, K2A, K4A, K6A, K7A, and W1A). Comparative analysis of CDR3 length distributions was performed and results were illustrated in Figure 2. As expected, the CDR3 length was less normally distributed in the ESRD patients compared to the healthy control individuals, which displayed nearly normal distribution. Statistical analysis showed that the difference in the CDR3 length distribution between the two groups was insignificant (*p* > .05).

Figure 2.The CDR3 length distribution of healthy control individuals and ESRD patients. (A) The CDR3 length distribution was displayed for each control individual (K1A, K2A, K4A, K6A, K7A, or W1A); (B) The CDR3 length distribution of each ESRD patient (A2A, A4A, A5A, A7A, A8A, A9A, R1A, R6A, R8A or R10A) was displayed; (C) Scatterplot analysis of distribution of CDR3 length in the ESRD (end-stage renal disease) patients versus normal control individuals. The distribution of the control group was more concentrated in contrast to that in the ESRD (end-stage renal disease) group, which was relatively dispersed; (D) Boxplot analysis of CDR3 length distribution in the ESRD (end-stage renal disease) group compared with the control group. The ESRD (end-stage renal disease) group showed substantially skewed distribution, whereas the control group was substantially normally distributed. *p* value was .1764.

**Figure:**
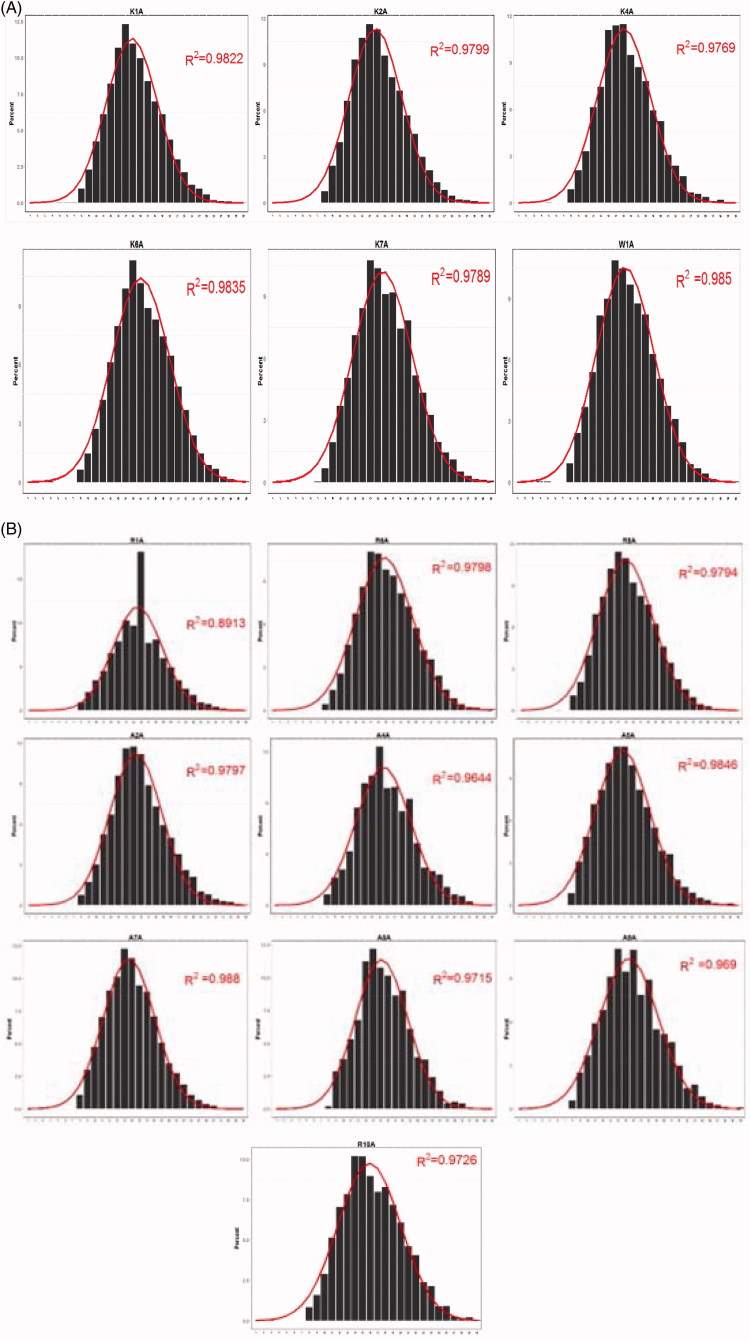

**Figure:**
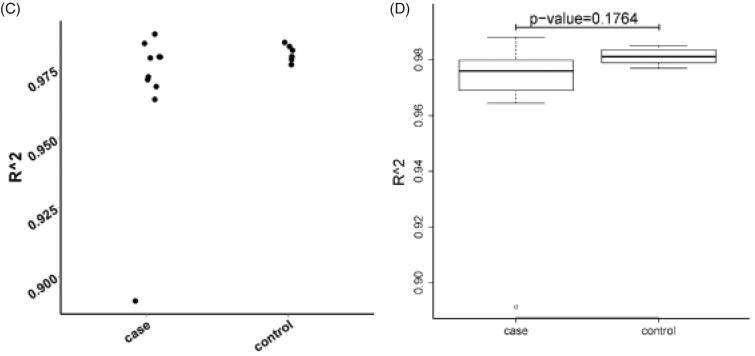

### Distinct usage frequency of V, D, and J gene segments in the BCR H chain CDR3 region

**Figure 3.**
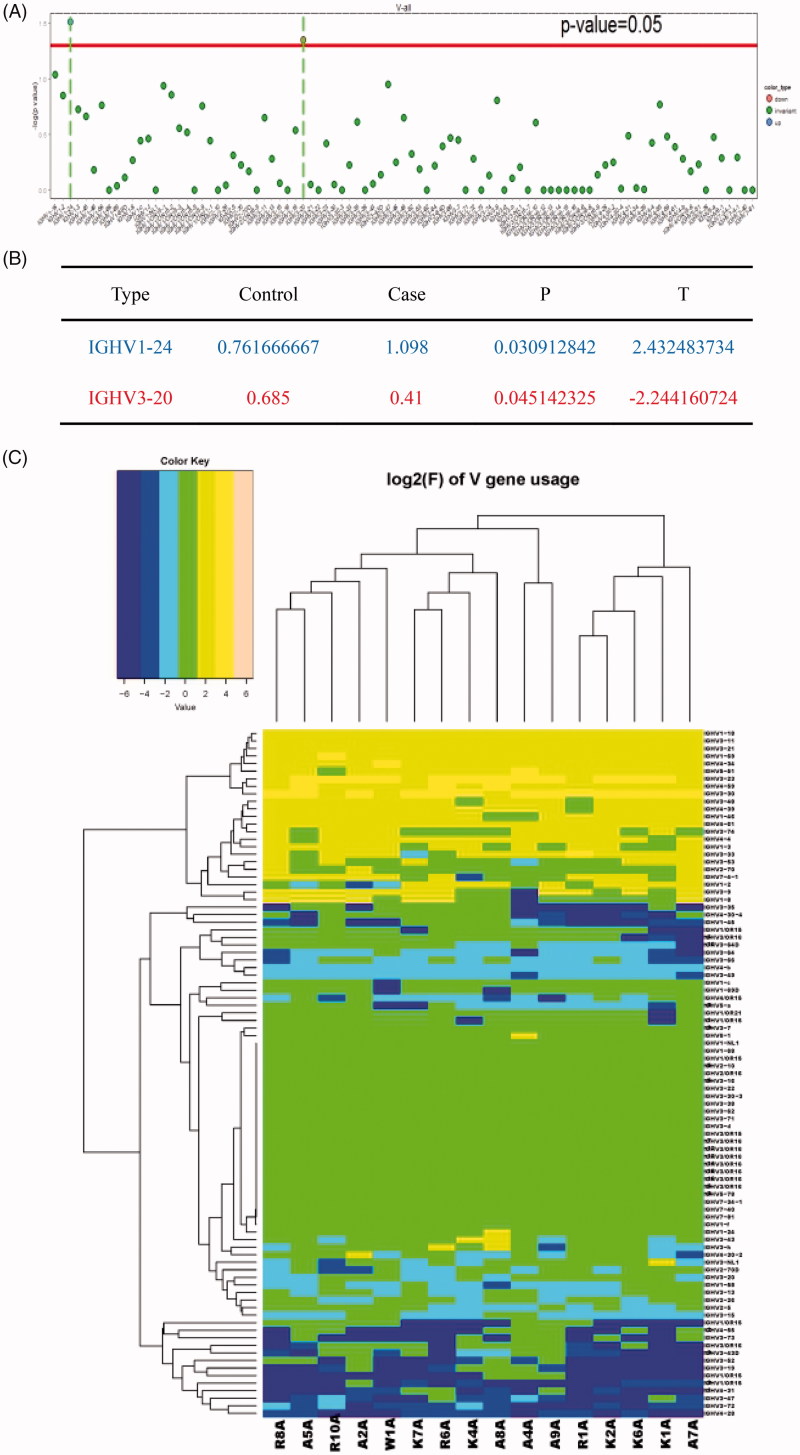
Usage frequency of V gene segments in the BCR H chain CDR3 region in ESRD patients versus healthy controls. (A) T-test was conducted in individual V gene between the ESRD (end-stage renal disease) group and control group for analysis of the distribution proportion. (B) Up-regulated gene was denoted in blue and down-regulated gene in red. In the T-test, values positive represented up-regulated genes, while those values negative indicated that genes were down-regulated; (C) The clustering heat map of V gene sub-types of the ESRD (end-stage renal disease) patients and healthy controls. For each sample, with a total of v of usage frequency and clustering, in order to show more samples of each corresponding differences in the frequency of changes among v sub-types, the frequency of the correlation coefficient for log2 do heat value.

We then determined differences in the usage frequency of the V, D, J gene segments in the BCR H chain CDR3 between the ESRD group and control group. T-test was conducted to analyze the usage frequency of the V, D, and J genes in 10 ESRD patients (A2A, A4A, A5A, A7A, A8A, A9A, R1A, R6A, R8A, and R10A) and six health control individuals (K1A, K2A, K4A, K6A, K7A, and W1A). Hierarchical clustering heat map was created to identify alterations in expression of studied individual gene fragments in the ESRD group compared with the healthy control group. IGHV1–24 gene was significantly up-regulated (*p* < .05), whereas IGHV3–30 was found to be down-regulated significantly (*p* < .05) in the ESRD group compared to the healthy control group (Figure 3).

**Figure 4.**
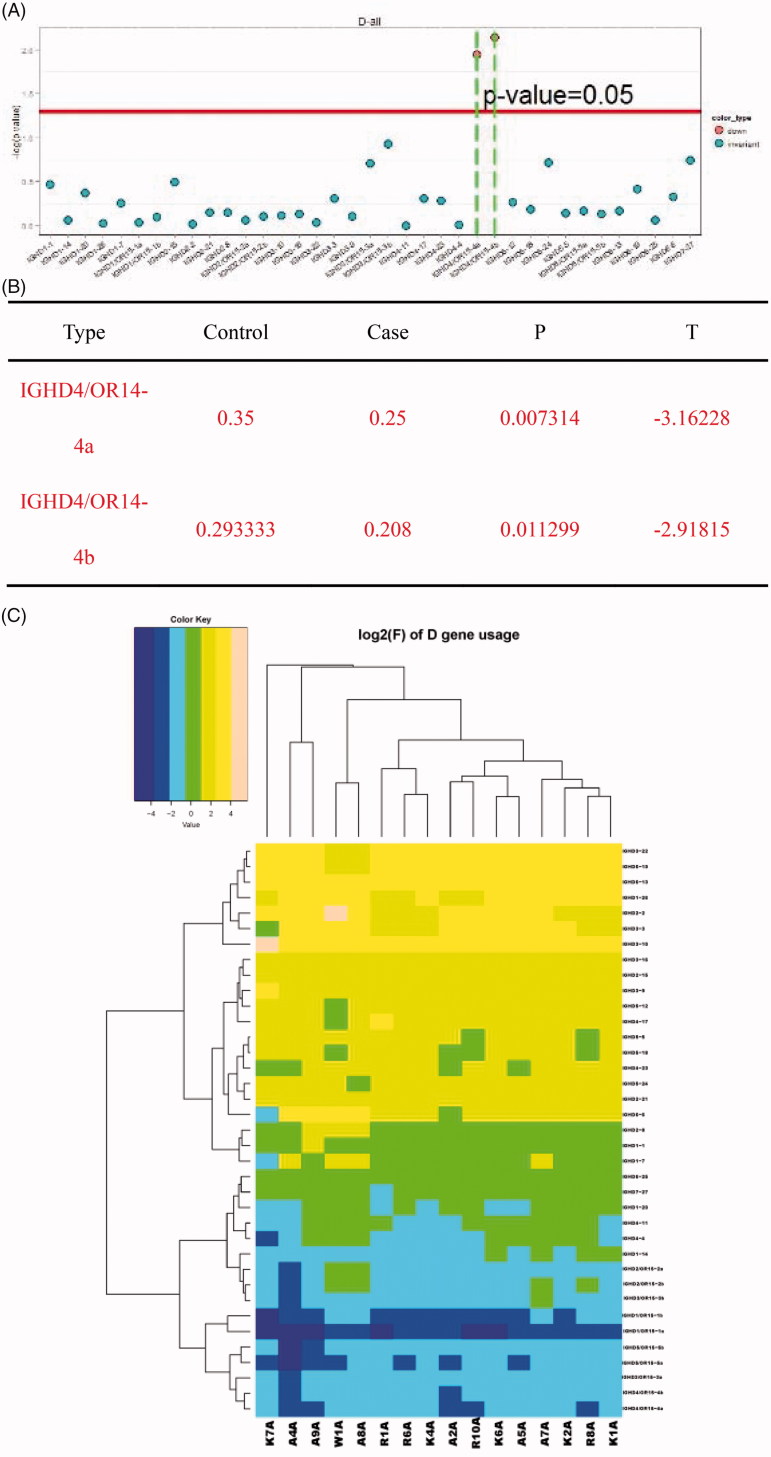
Usage frequency of D gene segments in the BCR H chain CDR3 region in ESRD patients versus healthy controls. (A) T-test was conducted in individual D gene between the ESRD (end-stage renal disease) group and control group for analysis of the distribution proportion. (B) Down-regulated genes were identified; (C) The clustering heat map of D gene sub-types of the ESRD (end-stage renal disease) patients and healthy controls.

Similarly, we created the distribution histogram of BCR heavy chain’s D region usage frequency, clustering heat map for D sub-genotype of each usage frequency, and performed T-test for distribution ratio of the D gene of 10 ESRD patients and six healthy controls. IGHD4/OR14–4a and IGHD4/OR14–4b with values negative by comparing the ESRD group with the healthy control group were down-regulated, and the differences were statistically significant (*p* < .05) (Figure 4).

**Figure 5.**
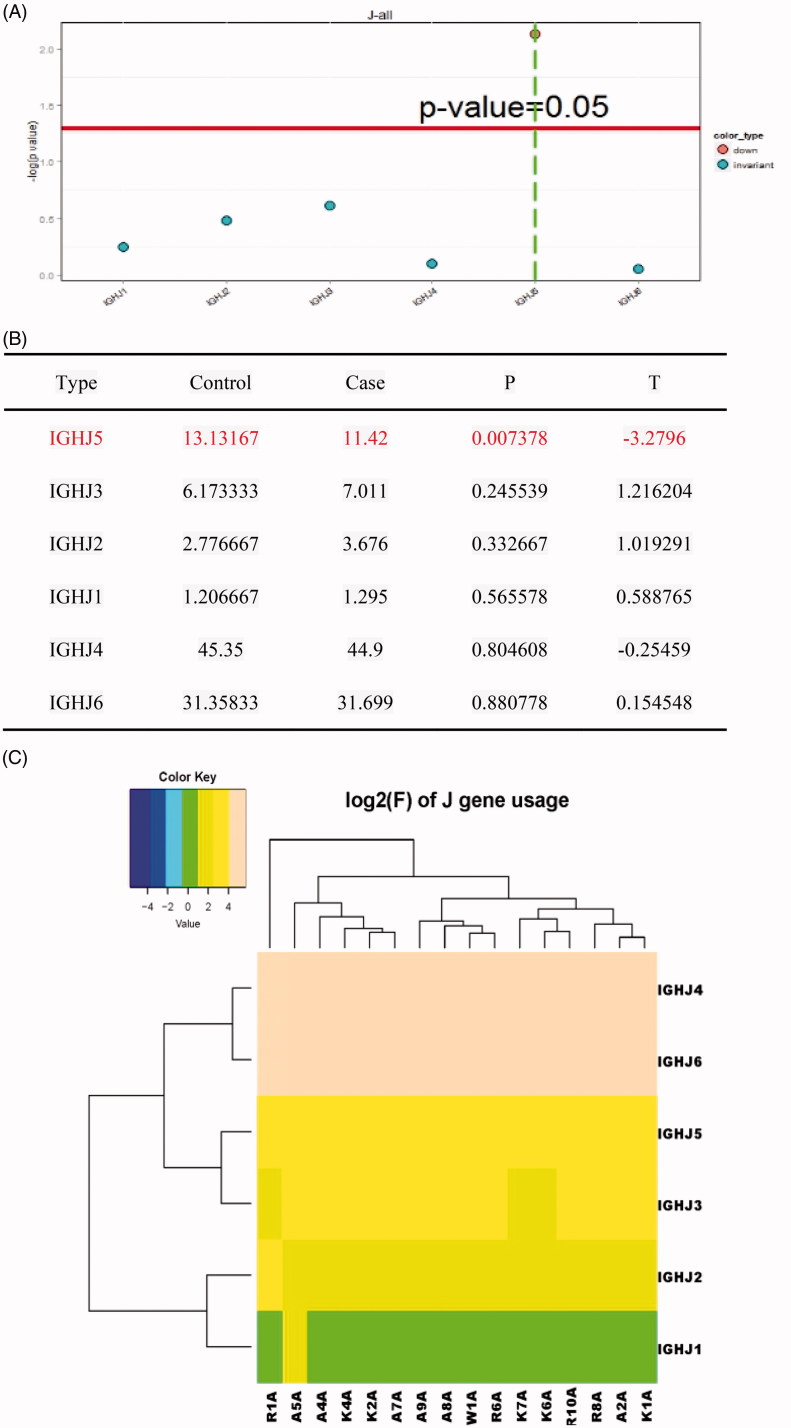
Usage frequency of J gene segments in the BCR H chain CDR3 region in ESRD patients versus healthy controls. (A) T test analysis for the J gene usage frequency in the ESRD (end-stage renal disease) patients versus control individuals; (B) Significantly down-regulated genes were type J5 (*p* < .05), and those genes with no significant changes were type J3, J2, J1, J4 and J6 (*p* > .05); (C) The clustering heat map of J gene sub-types of the ESRD (end-stage renal disease) patients and healthy controls.

We also generated distribution histogram of BCR heavy chain’s J region usage frequency, and J sub-genotype of each frequency clustering heat map. T-test for distribution ratio of the J gene of 10 ESRD patients and six controls allowed us to visually identify the expression of individual genes in patients with ESRD group and the normal control group. IGHJ5 was significantly down-regulated in the ESRD group in contrast to the healthy control group (*p* < .05), whereas no significant alteration in expression of the IGHJ1, IGHJ2, IGHJ3, and IGHJ4 genes were observed between the two groups (Figure 5).

### The combinations of the BCR H chain V and J genes

**Figure 6.**
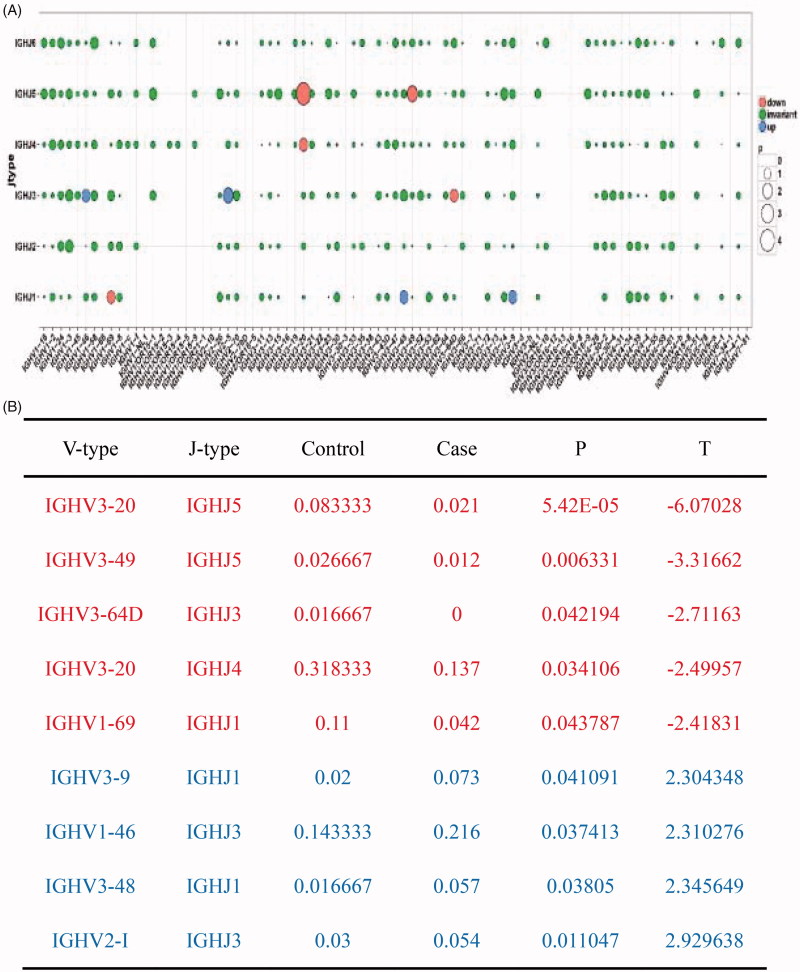
Comparative analysis of combinations of IGHJ and IGHV genes in ESRD patients versus control individuals. (A) Up-regulated genes were denoted in circle 2, while down regulated genes were marked in circle 3 and 4. There was no significant change in circle 1 and 0, a combination of circle 2, 3 and 4; (B) More combinations of IGHJ and IGHV were observed in ESRD (end-stage renal disease) group compared to the control group.

To comparatively analyze the combinations of BCR H chain V and J genes in the two group, a bubble chart was generated, and T-test was performed to examine the distribution ratio of combinations in the ESRD patients (A2A, A4A, A5A, A7A, A8A, A9A, R1A, R6A, R8A, and R10A) and healthy controls (K1A, K2A, K4A, K6A, K7A, and W1A). As shown in Figure 6, the ESRD group showed greater changes in V–J combinations compared with the normal control group. Of the V–J combinations, five combinations (IGHV3–20, IGHJ5; IGHV3–49, IGHJ5; IGHV3–64D, IGHJ3; IGHV3–20, IGHJ4; and IGHV1–69, IGHJ1) were significantly down-regulated (*p* < .05), while four combinations (IGHV3–9, IGHJ1; IGHV1–46, IGHJ3; IGHV3–48, IGHJ1; and IGHV2-I, IGHJ3RD) were significantly up-regulated in the ESRD group compared to the healthy control group (*p* < .05).

### TOP 20 most frequent BCR H chain V gene segments

**Figure 7.**
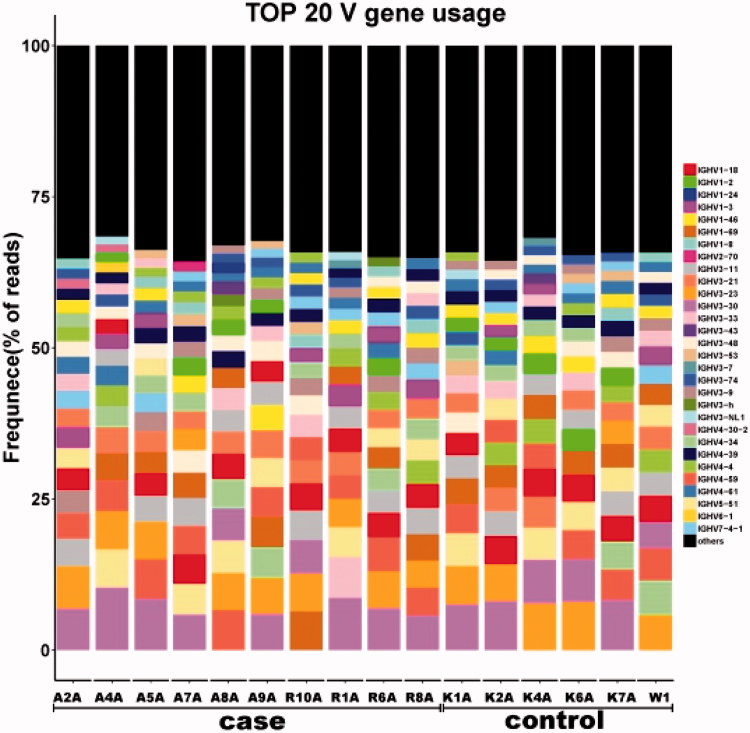
Identification of the most frequently used BCR H chain V gene segments in the ESRD and control groups. The Y axis represented the top 20 most frequent usage of BCR H chain V gene subtypes, and the remaining V sub-types were indicated in black.

The frequency usage of the BCR H chain V gene segments were analyzed in each sample from the ESRD and control groups. Top 20 most frequent V gene segments or subtypes in the ESRD patients (A2A, A4A, A5A, A7A, A8A, A9A, R1A, R6A, R8A, and R10A) and healthy controls (K1A, K2A, K4A, K6A, K7A, and W1A) were illustrated in Figure 7.

### Shared amino acid sequences in the BRC H chain CDR3 region by the ESRD and healthy groups

In analysis of the amino acid sequences, we found that three amino acid sequences (AR, FDY, and MDV) were shared by all participants from the ESRD group (*n* = 10) and the normal control (NC) group (*n* = 6). The clone numbers of the shared CDR3 amino acid sequences in each ESRD patient along with the total numbers of all CDR3 clones were listed in Table 2. In parallel, Table 3 showed the clone numbers of the common CDR3 amino acid sequences in each health individual in the control group.

**Table 2.** The Clone numbers of the CDR3 amino acid sequences in each ESRD patient.

CDR3	A2A	A4A	A5A	A7A	A8AA	A9A	R10A	R1A	R6A	R8A
AR	2990	2	378	226	32	4027	58	101	2	89
FDY	8	7	45	31	2	3	6075	5	98	24
MDV	59	18	88	89	76	31	31	32	127	91
Total AA CDR3	12922736	10696076	12608164	12636581	10666652	10274739	8524525	10652390	10981099	8916519

**Table 3.** The Clone numbers of the CDR3 amino acid sequences in each healthy individual.

CDR3	K1A	K2A	K4A	K6A	K7A	W1A
AR	262	137	97	2	80	3
FDY	42	37	69	3	17	12
MDV	69	60	13	1	12	146
Total AA CDR3	12584061	12249709	10600111	439959	11778081	10575248

## Discussion

In this study, we sequenced the BCR H chain CDR3 repertoire of peripheral B cells from 10 patients diagnosed with ESRD and six health control individuals using NGS technique, and analyzed composition and variation of each BCR H chain CDR3 sequences. The major novel findings were summarized as follows: (1) The ESRD patients exhibited greater extent of clonal expansion of the B cells in contrast to the healthy control individuals; (2) The ESRD patients displayed skewed usage of the BCR CDR3 V, D, J, and V–J gene segments, whereas the NC group appeared to be normally distributed; (3) Dysregulation of the BCR H chain CDR3 of V, D, J, and V–J combinations subfamily genes were observed in relation to ESRD, suggesting relationship between the BCR CDR3 repertoire and the development of ESRD disease.

ESRD is simultaneously associated with immune activation, which is featured by systemic inflammation and immune deficiency [18]. During the development of B lymphocytes, V, D, and J gene segments of BCR are rearranged and different number of nucleotides can either randomly insert into V–J segments or V-D-J segments. Oppositely, different number of nucleotides can randomly delete in V–J segments or V-D-J segments. Through the insertion or deletion, a highly diversity variable CDR3 region can be generated in ways of differences in length and amino acid sequences of the BCR CDR3. In fact, CDR3 sequences determine a unique BCR clone type, and for which the clonal diversity of B lymphocytes by detecting the length of CDR3 can be evaluated. In our study, we compared normality of CDR3 length distribution between ESRD patient and NC groups and observed head of distribution tended to be skewed distribution in the ESRD group with normal distribution in the NC group. In comparative analysis with Shannon entropy and HEC of each sample between ESRD patients and NC group, differences between the two groups were observed, but not statistically significant, possibly due to a small sample size used in this study. In T test for frequency usage of BCR H chain V, D, J, and V–J segments, we identified five up-regulated genes: IGHV1–24, V–J combination (IGHV3–9, IGHJ1), V–J combinations (IGHV1–46, IGHJ3), V–J combination (IGHV3–48, IGHJ1), V–J combination (IGHV2-I, IGHJ3); and nine down-regulated genes: IGHV3–20, IGHD4/OR14–4a, IGHD4/OR14–4b, IGHJ5, V–J combination (IGHV3– 20, IGHJ5), V–J combinations (IGHV3–49, IGHJ5), V–J combinations (IGHV3–64D, IGHJ3), V–J combinations (IGHV3–20, IGHJ4), V–J combinations (IGHV1–69, IGHJ1). These abnormal expression of BCR H chain CDR3 of V, D, J, and V–J combinations sub-family gene may be involved in the development of ESRD disease. In addition, these up-regulated genes IGHV1–24, V–J combination (IGHV3–9, IGHJ1), V–J combinations (IGHV1–46, IGHJ3), V–J combinations (IGHV3–48, IGHJ1), V–J combination (IGHV2-I, IGHJ3) may be associated with BCR-specific clonal proliferation of B lymphocytes and undermining BCR diversity. As the BCR diversity plays an essential role in effective immune response in healthy population, the high diversity in subtypes of immune proteins is expected to have more effective immune response against pathogens. However, if the fewer subtypes in the immune proteins indicate low diversity and less or even ineffectiveness in responding to pathogens and more susceptible to disease. It was of note in our study, the following five genes including IGHV3–20, IGHD4/OR14–4a, IGHD4/OR14–4b, IGHJ5, V–J combination (IGHV3–20, IGHJ5), V–J combination (IGHV3–49, IGHJ5), V–J combinations (IGHV3–64D, IGHJ3), V–J combination (IGHV3–20, IGHJ4), V–J combinations (IGHV1–69, IGHJ1) were down-regulated, and these may be involved in inhibiting BCR certain specific clonal proliferation of B lymphocytes. These findings were consistent with the previous reports that the B lymphocytes in patients with ESRD were significantly reduced.

In conclusion, we developed an assay using HTS technology to assess variations in B-cell repertoires at sequence-level resolution in ESRD. Our results demonstrated a direct evidence on the correlation between BCR H chain CDR3 repertoire and ESRD. In-depth studies are needed to better understand the role of the BCR repertoire in immune responses, autoimmunity and alloreactivity in the development of ESRD. Until now, few articles about B-cell receptor heavy chain repertoire in patients with end-stage renal disease by high-throughput sequencing has been reported. The limitation of our article is that the sample size is small. So larger sample research needs to continue.
